# Regulation of Arabidopsis Matrix Metalloproteinases by Mitogen-Activated Protein Kinases and Their Function in Leaf Senescence

**DOI:** 10.3389/fpls.2022.864986

**Published:** 2022-04-08

**Authors:** Hongjiao Wu, Qi Si, Jianmin Liu, Liuyi Yang, Shuqun Zhang, Juan Xu

**Affiliations:** ^1^State Key Laboratory of Plant Physiology and Biochemistry, College of Life Sciences, Zhejiang University, Hangzhou, China; ^2^Interdisciplinary Plant Group, Division of Biochemistry, University of Missouri, Columbia, MO, United States

**Keywords:** MAPK cascade, matrix metalloproteinase, leaf senescence, *Arabidopsis*, transcription regulation

## Abstract

Leaf senescence is a developmentally programmed cell death process that is influenced by a variety of endogenous signals and environmental factors. Here, we report that MPK3 and MPK6, two Arabidopsis mitogen-activated protein kinases (MAPKs or MPKs), and their two upstream MAPK kinases (MAPKKs or MKKs), MKK4 and MKK5, are key regulators of leaf senescence. Weak induction of constitutively active MAPKKs driven by steroid-inducible promoter, which activates endogenous MPK3 and MPK6, induces leaf senescence. This gain-of-function phenotype requires functional endogenous MPK3 and MPK6. Furthermore, loss of function of both *MKK4* and *MKK5* delays leaf senescence. Expression profiling leads to the identification of matrix metalloproteinases (MMPs), a family of zinc- and calcium-dependent endopeptidases, as the downstream target genes of MPK3/MPK6 cascade. MPK3/MPK6 activation-triggered leaf senescence is associated with rapid and strong induction of *At3-MMP* and *At2-MMP*. Expression of Arabidopsis MMP genes is strongly induced during leaf senescence, qualifying them as senescence-associated genes (SAGs). In addition, either constitutive or inducible overexpression of *At3-MMP* is sufficient to trigger leaf senescence. Based on these findings, we conclude that MPK3/MPK6 MAPK cascade and *MMP* target genes further downstream are involved in regulating leaf senescence in *Arabidopsis.*

## Introduction

Senescence at the final stage of plant growth season or life cycle is a programmed cell death process that is influenced by both endogenous developmental programs and environmental factors (Gan and Amasino, 1997; Lim et al., 2007; Woo et al., 2013). Leaf yellowing as a result of the disruption in photosynthesis and the loss of chlorophyll due to the breakdown of the structural integrity of chloroplasts is a typical phenotypic symptom of leaf senescence. This is accompanied by other dramatic changes in cellular metabolisms, such as degradation of membrane lipids, proteins, nucleic acids, and other macromolecules (Gan and Amasino, 1997; Lim et al., 2007). Nutrients released from the senescing leaves are recycled and used by younger growing leaves, developing seeds, or storage tissues to ensure an optimal condition for the next generation or growing season (Gan and Amasino, 1997; Guo and Gan, 2005; Lim et al., 2007; Woo et al., 2016). In addition to age, the onset and progression of leaf senescence are influenced by various internal signals including abscisic acid, salicylic acid, ethylene, and jasmonate, as well as environmental stresses such as darkness, extreme high or low temperature, nutrient deficiency, oxidative stress, and pathogen infections (Gan, 2003; Lim et al., 2007). Although many genes have been identified as senescence-associated genes (*SAGs*), their exact functions in the senescence remain largely unclear (Li et al., 2012, 2014).

Mitogen-activated protein kinase (MAPK or MPK) cascades are major pathways by which extracellular stimuli are transduced into cellular responses in eukaryotic cells (Ichimura et al., 2002; Pedley and Martin, 2005; Pitzschke et al., 2009; Andreasson and Ellis, 2010; Rodriguez et al., 2010; Tena et al., 2011; Xu and Zhang, 2015; Zhang et al., 2018). A basic MAPK cascade is composed of three interconnected kinases, a MAPK (MPK), which is activated by its upstream MAPK kinase (MAPKK, MKK, or MEK), via phosphorylation of the TXY activation motif. MAPKK activity is in turn regulated by the topmost member of the module, MAPKK kinase (MAPKKK, or MEKK), via phosphorylation. A MAPKKK receives signals from receptors/sensors either directly or indirectly. In *Arabidopsis*, there are 20 MAPKs, 10 MAPKKs, and approximately 60 putative MAPKKKs (Ichimura et al., 2002). MPK3 and MPK6, two Arabidopsis MAPKs with the highest homology, share two redundant upstream MAPK kinases, MKK4 and MKK5 (Wang et al., 2007; Su et al., 2017, 2018). In plant defense, MAPKKK3 and MAPKKK5 function redundantly upstream of MKK4/MKK5-MPK3/MPK6 in plant PAMP-triggered immunity (Bi et al., 2018; Sun et al., 2018). In plant development, YDA is upstream of the MKK4/MKK5-MPK3/MPK6 module in plant inflorescence architecture and stomatal development (Bergmann et al., 2004; Wang et al., 2007; Meng et al., 2012). Several reports have revealed the important roles of MAPK signaling cascade in regulating leaf senescence. MKK9-MPK6 cascade was shown to play a positive role in regulating leaf senescence in *Arabidopsis* (Zhou et al., 2009). MPK6-WRKY6-NPR1 and MPK6-EIN2-EIN3-ORE9 modules were implicated in SA- and MeJA-induced leaf senescence, respectively (Chai et al., 2014; Zhang et al., 2016). MAPKKK18-MKK3-MPK1/2/7, another MAPK cascade, involved in ABA-triggered leaf senescence (Matsuoka et al., 2015). Recently, MKK4/MKK5-MPK1/MPK2 cascade was reported to regulate SA-induce leaf senescence via phosphorylation of NPR1 (Zhang et al., 2020). These findings suggest a complex regulation network involved the MAPK signaling network during leaf senescence.

Matrix metalloproteinases (MMPs) are a family of zinc- and calcium-dependent endopeptidase (Rawlings et al., 2010). As a well-known proteolytic enzyme in animals, MMPs are involved in extracellular matrix remodeling, cell migration, cell proliferation, adhesion, and cellular signaling by limited proteolytic processing of their substrate proteins (Sternlicht and Werb, 2001; Overall, 2002; Parks et al., 2004; Page-McCaw et al., 2007). MMPs have also been identified in plants (Pak et al., 1997). Based on their expression patterns, plant MMPs have been implicated in plant growth and development (Delorme et al., 2000; Liu et al., 2001; Frick and Schaller, 2002; Ratnaparkhe et al., 2009; Schiermeyer et al., 2009; Zimmermann et al., 2016; Das et al., 2018). Soybean *SEMP1/Gm1-MMP* was shown to be expressed only in mature leaves, suggesting a role in tissue remodeling during leaf expansion (Pak et al., 1997). In cucumber, *Cs1-MMP* was found to be associated with senescence and cell death in cotyledon development (Delorme et al., 2000). A subtilisin-like proteinase p69b was identified as a substrate of tomato *Sl-MMPs* in cell death control (Zimmermann et al., 2016). More recently, Rice *OsMMP1* was reported to play pleiotropic roles in plant development and symplastic-apoplastic transport by modulating cellulose and callose depositions (Das et al., 2018). In *Arabidopsis*, the MMP family consists of five members named *At1-MMP* to *At5-MMP* (Maidment et al., 1999). All five *At-MMP* genes display distinct tissue/organ development-specific expression patterns, suggesting differential physiological functions for each enzyme (Maidment et al., 1999; Flinn, 2008).

In this study, we found that a weak and long-lasting activation of MPK3/MPK6 after a low-level induction of the constitutively active MAPKKs upstream (MKK4, MKK5, and their tobacco ortholog NtMEK2) is sufficient to trigger leaf senescence, which is associated with *At3-MMP* and *At2-MMP* gene activation. In contrast, loss of function of both *MKK4* and *MKK5* delays leaf senescence. Arabidopsis *MMP* gene expression is strongly induced during leaf senescence, qualifying them as senescence-associated genes (*SAG*s). Over-expression of *At3-MMP*, either constitutively or under the control of an inducible promoter, is sufficient to trigger leaf senescence. Collectively, this study reveals a signaling pathway involving MPK3/MPK6 cascade and *MMP* target genes in leaf senescence in *Arabidopsis thaliana*.

## Materials and Methods

### Plant Materials and Growth Conditions

After surface-sterilized and vernalization at 4°C for 3–5 days, seeds were sown in half-strength Murashige and Skoog (MS) medium with 0.45% Phytagar and grown in a growth chamber at 22°C with continuous light (80 μE/m^–2^s^–1^). Seven-day-old seedlings were transplanted to soil and grown at 22°C and a 14-h-light/10-h-dark cycle. Col-0 ecotype was used as the wild type. T-DNA insertion mutants including *at1-mmp* (SALK_205145C), *at2-mmp* (SM_3_5305), *at3-mmp* (SM_3_28404), *at4-mmp* (GABI_075C07), *at5-mmp* (SAIL_390_c06), *mpk3-1*(SALK_151594), *mpk6-2* (SALK_073907), *mkk7* (SM_3_21961), and *mkk9* (SAIL_60_H06) were obtained from the Arabidopsis Biological Resource Center (ABRC). The *mkk4* and *mkk5* single tilling mutants (Zhao et al., 2014) were backcrossed with Col-0 to remove the *er-105* mutant allele and then were crossed to generate a *mkk4 mkk5* double mutant (Li et al., 2018). Transgenic plant *GVG:NtMEK2*^DD^** (abbreviated as *DD*), in which dexamethasone (DEX)-inducible promoter-driven constitutively active *NtMEK2^DD^* transgene, was previously described (Yang et al., 2001; Ren et al., 2002). *DD mpk3* (Wang et al., 2007) and *DD mpk6* (Liu and Zhang, 2004) were generated by genetic cross between *DD* and *mpk3-1*, *DD* and *mpk6-2*, respectively. The *mmp2 mmp3* double mutant, *mmp2 mmp3 mmp5* triple mutant, *at1;2;3;4;5-mmp* pentuple mutant, and *mkk7 mkk9* double mutant were generated by genetic cross and homozygous mutant plants were identified using T-DNA border primers and gene-specific primers (listed in Supplementary Table 1).

To generate DEX-inducible promoter-driven *At-3MMP* construct (*GVG:At3-MMP-dHA*) and constitutive overexpression *35S:At3-MMP-dHA* construct, we amplified the full-length *At-3MMP* cDNA fragment using primers LP1/RP1 and LP2/RP2 and cloned the PCR fragment into a modified pBlueScript II vector with a double HA tag at the *3*′-end. The *3MMP-dHA* fragment was subsequently cloned into the *Xho*I/*Spe*I sites of the pTA7002 vector and a modified pBI121 vector with 35S double enhancer and *Xho*I/*Spe*I restriction sites (pBId vector), respectively. These two binary vectors were transformed into *Agrobacterium tumefaciens* strain GV3101. Arabidopsis transformation was performed by the floral dip procedure (Clough and Bent, 1998), and transformants were identified by screening for hygromycin (pTA7002 vector) or kanamycin (pBId vector) resistant T1 seedlings. Independent lines with *At-3MMP* transgene induction or expression were identified based on immunoblot analysis. From these transformations, two independent lines with a single copy of T-DNA insertion (based on the 3:1 segregation of antibiotic resistance in T2 progeny) were isolated, and homozygote transgenic plants were further identified in the progeny based on segregation of antibiotic resistance.

### Leaf Senescence Assays

Leaves from 4-week-old plants were used for the leaf senescence assay. For detached leaf senescence analysis, fully expanded leaves (from the fifth to eighth leaf position) were detached and their petioles were inserted into 0.6% agar plates with DEX or EtOH solvent with the adaxial side facing up. The plates were kept under light (60 μE/m^–2^s^–1^) at 22°C. For observation of leaf senescence in whole plants, 4-week-old soil-grown plants were sprayed with 30 μM DEX. Whole plants were photographed at indicated times after treatment.

### Measurement of Chlorophyll Content and Photochemical Efficiency

Chlorophyll was extracted with 80% (v/v) acetone from detached leaves (Arnon, 1949). Chlorophyll contents were measured at 645 and 663 nm and chlorophyll concentrations were calculated as (20.2 × *A*_645_ + 8.02 × *A*_663_)/g fresh weight. Maximal quantum yield of PSII photochemistry (F_*v*_/F_m_) was determined using a PAM 2000 portable chlorophyll fluorimeter in the dark-adapted leaf samples.

### RNA Extraction and Real-Time Quantitative PCR Analysis

Total RNA was extracted using Trizol reagent (Invitrogen). RNA concentration was measured using a NanoDrop (Model 2000C). After an additional ethanol precipitation and DNase treatment, 1 μg of total RNA was used for reverse transcription. Quantitative PCR was conducted using a real-time PCR machine (Eppendorf, Germany). *EF1*α was used for internal control. The relative gene expression was calculated using the double ΔCt method. The absolute copy numbers were calculated based on the standard curve for each *MMP* gene for better assessment of the potential contribution of each *MMP* gene. The primers pairs used for qPCR are listed in Supplementary Table 1.

### Immunoblot Analysis

Total protein was extracted from leaf tissues by grinding with 3x volume of 1xSDS sample buffer [60 mM Tris/HCl, pH 6.8, 10% (v/v) glycerol, 1% (w/v) SDS] followed by boiling for 15 min. Protein extracts for immunoblot detection of phosphorylated MAPKs were prepared by grinding with extraction buffer [100 mM HEPES, pH 7.5, 5 mM EDTA, 10 mM DTT, 10 mM Na_3_VO_4_, 10 mM NaF, 50 mM β-glycerophosphate, 1 mM phenylmethylsulfony fluoride (PMSF), 5 μg/mL antipain, 5 μg/mL aprotinin, 5 μg/mL leupeptin, and 10% (v/v) glycerol]. Protein concentration was determined by using the Bio-Rad protein assay kit with BSA as a standard. Total proteins (10 μg) were separated in 10% SDS-PAGE gels. At-3MMP and DD protein induction were detected by immunoblot analysis using anti-HA (Sigma, dilution 1:10,000) and anti-FLAG (Sigma, dilution 1:10,000), respectively. Activation of MPK3 and MPK6 was detected by using anti-pTEpY (Cell Signaling, dilution 1:3,500). The blots were incubated with horseradish peroxidase-conjugated goat anti-mouse (for anti-HA and anti-FLAG) or goat anti-rabbit (for anti-pTEpY) secondary antibodies. Coomassie brilliant blue staining of duplicated gels was used to confirm equal loading.

### Statistical Analysis

At least two independent repetitions were performed for experiment with multiple time points. For single time point experiments, at least three independent repetitions were done. Results from one of the independent repeats that gave similar results were shown. Two-way ANOVA assay was used to determine whether the difference between two groups of data at a specific time point is statistically significant (*P* < 0.05). Statistically different data groups are indicated by using asterisks placed above the columns in the graphs.

### Accession Numbers

Sequence data from this article can be found in the Arabidopsis Genome Initiative or GenBank/EMBL databases under the following accession numbers: *At1-MMP* (At4G16640), *At2-MMP* (At1G70170), *At3-MMP* (At1G24140), *At4-MMP* (At2G45040), *At5-MMP* (At1G59970), *MPK3* (At3G45640), *MPK6* (At2G43790), *MKK4* (At1G51660), *MKK5* (At3G21220), *EF1*α (At5G60390, and *SAG12* (At5G45890).

## Results

### Weak Induction of Constitutively Active MAPK Kinases Leads to MPK3- and MPK6-Dependent Leaf Senescence

**FIGURE 1:**
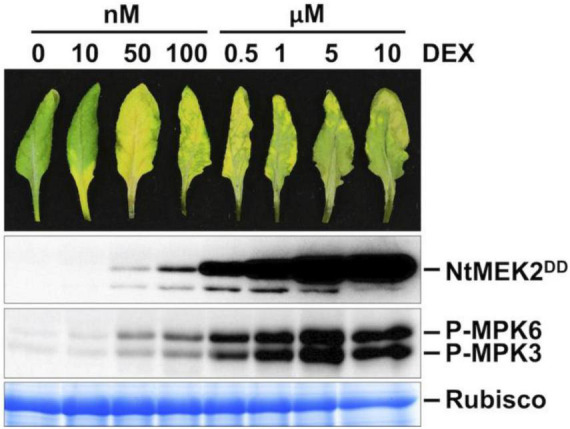
Weak induction of constitutively active MAPKK promotes leaf senescence. Treatment of *DD* plants with different concentrations of DEX can control the amplitude of MPK3/MPK6 phosphorylation/activation. Fully expanded leaves from 4-week-old soil-grown plants were detached and their petioles were inserted into 0.6% agar medium with different concentrations of DEX. Photos were taken at 3 days after DEX treatment (first panel). Leaves treated with DEX at 0.5 μM or above showed rapid cell death and stayed green. Leaf samples were collected at 1 day after DEX treatment. DD protein induction was detected by immunoblot assay using an anti-flag antibody (second panel). MAPK activation was detected by immunoblot assay using an anti-pTEpY antibody (third panel). Coomassie brilliant blue staining of a duplicated gel was used to confirm equal loading (fourth panel).

**FIGURE 2:**
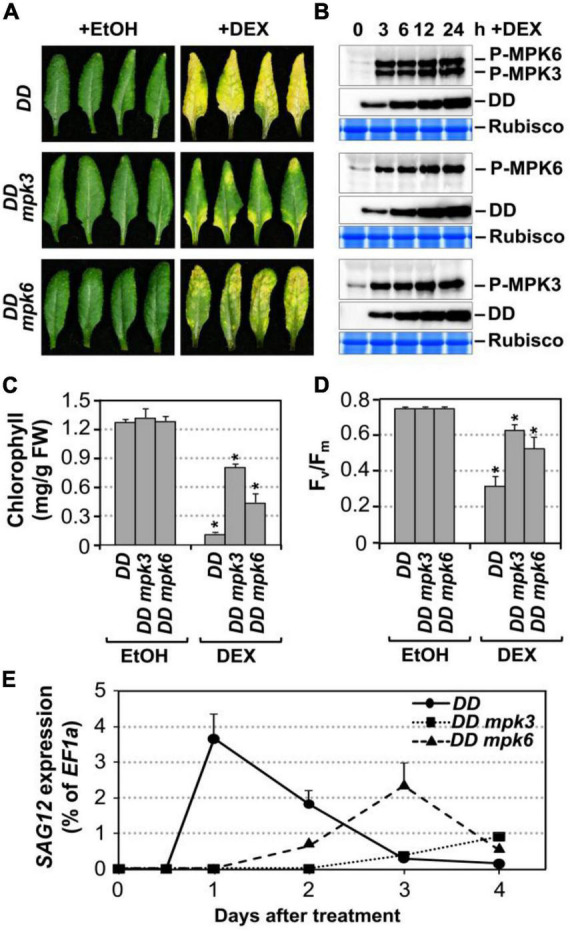
Leaf senescence in gain-of-function *DD* plant is dependent on endogenous MPK3 and MPK6. **(A)** Fully expanded leaves from 4-week-old *DD*, *DD mpk3*, *DD mpk6* plants were detached and their petioles were inserted into 0.6% agar medium with DEX (100 nM) or EtOH (solvent control). Photos were taken 4 days after treatment. **(B)** MAPK activation (top) and DD protein induction (middle) in *DD*, *DD mpk3*, and *DD mpk6* plants after DEX treatment were detected by immunoblot analysis using anti-pTEpY and anti-Flag antibody, respectively. Coomassie brilliant blue staining of duplicated gels were used to confirm equal loading (bottom). **(C,D)** Measurement of chlorophyll content **(C)** and F_*v*_/F_m_ **(D)** in leaves shown in **(A)**. Asterisks indicate a significant difference (*P* ≤ 0.001). Values are means ± SD, *n* = 10. **(E)** RT-qPCR analysis of *SAG12* expression in leaves at indicated times after DEX treatment. Gene expression was calculated using the double ΔCt method and *EF1*α was used as a reference. Values were means ± SD, *n* = 3.

To understand the function of MPK3 and MPK6 in *Arabidopsis* and their orthologs in other plant species, we previously generated conditional gain-of-function systems by expressing the constitutively active MAPKK (Arabidopsis MKK4 and MKK5, and tobacco NtMEK2) variants under the control of a steroid-inducible promoter (Yang et al., 2001; Ren et al., 2002). In this study, *DD* plants refer specifically to *GVG:NtMEK2*^DD^** transgenic Arabidopsis (abbreviated as *DD* for the substitution of two Ser/Thr residues in the activation loop of MAPKK with Asp to make it constitutively active). Full induction of transgene expression after dexamethasone (DEX) treatment induces high-level activation of endogenous MPK3/MPK6 in *Arabidopsis*, which triggers a hypersensitive response (HR)-like cell death (Figure 1; Yang et al., 2001; Ren et al., 2002). However, when these plants were treated with lower concentrations of DEX (equal or less than 100 nM), the plants showed a leaf-yellowing phenotype similar to senescence (Figure 1). Similarly, lower concentrations of DEX treatment of *GVG:AtMKK4*^DD^** and *GVG:AtMKK5*^DD^** Arabidopsis plants also lead to leaf-yellowing phenotype (Supplementary Figure 1). This phenotype is dependent on functional *MPK3* and *MPK6*. As shown in Figure 2A, in either *mpk3* or *mpk6* single mutant background, the gain-of-function *DD*-induced leaf yellowing phenotype was attenuated.

To confirm that the leaf yellowing phenotype in *DD* plants treated with low concentrations of DEX is indeed a senescence process, we measured typical senescence-associated physiological markers including chlorophyll content, photochemical efficiency of photosystem II (monitored as F_*v*_/F_m_), and the transcript levels of *SAG12*, a widely used molecular marker for leaf senescence (Noh and Amasino, 1999; Pontier et al., 1999). As shown in Figure 2C, the chlorophyll content decreased by approximately 92, 42, and 66% in the *DD*, *DD mpk3*, and *DD mpk6* plants, respectively. Similarly, F_*v*_/F_m_ value reduced by ∼56% in the *DD* plants at 4 days after DEX treatment. In contrast, in *mpk3* or *mpk6* mutant background, *DD*-induced F_*v*_/F_m_ reduction was significantly attenuated (Figure 2D). Furthermore, *SAG12* expression was strongly induced in *DD* plants, while the induction of *SAG12* was partially compromised in *DD mpk3* and *DD mpk6* plants (Figure 2E). Taken together, these results suggested *DD*-induced leaf yellowing is a senescence process, which is dependent on functional downstream MPK3 and MPK6.

To further confirm that MPK3 and MPK6 were responsible for the induction of leaf senescence in *DD* plants, we measured the activation of MPK3/MPK6 using phospho-specific pTEpY antibody in *DD*, *DD mpk3*, and *DD mpk6* plants. Immunoblot analysis using anti-flag antibody showed comparable DD protein induction in all three genotypes (Figure 2B). The delayed leaf senescence in *DD mpk3* and *DD mpk6* plants demonstrated that leaf senescence induction by *DD* required the activation of endogenous MPK3 and MPK6. The senescence phenotype in *DD mpk3* leaves was much less severe than that in *DD mpk6* leaves in comparison to the *DD* control, suggesting that MPK3 plays a more important role than MPK6 in this process.

### Leaf Senescence Was Delayed in *mkk4 mkk5* Double Mutant

**FIGURE 3:**
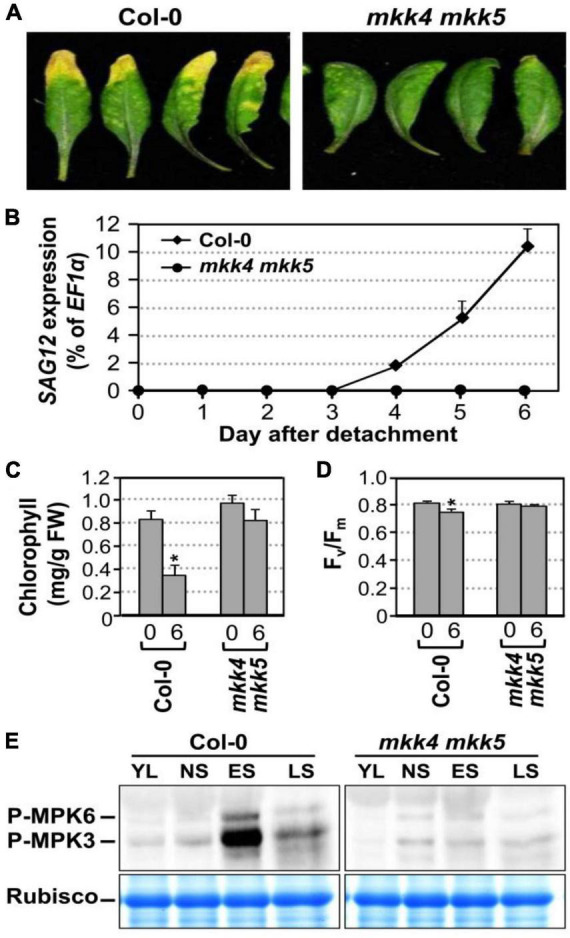
MKK4 and MKK5 play redundant function in leaf senescence. **(A)** Leaf senescence phenotype of detached leaves of wild type and *mkk4 mkk5* double mutants. Fully expanded leaves from 4-week-old soil-grown plants were detached and their petioles were inserted to 0.6% agar medium under continuous light. Photos were taken 6 days after detachment. **(B)** RT-qPCR analysis of the transcript levels of *SAG12* in wild type and *mkk4 mkk5* double mutants at indicated times. The expression of *EF1*α was used as an internal reference. Values are means ± SD, *n* = 3. **(C,D)** Quantitative analysis of chlorophyll contents **(C)** and F_*v*_/F_m_ **(D)** in leaves shown in **(A)**. Values are means ± SD, *n* = 10. **(E)** Phosphorylation/activation of MPK3/MPK6 induced by age-dependent leaf senescence was detected by immunoblot analysis using anti-pTEpY antibody (upper). Equal loading of proteins was confirmed by Coomassie brilliant blue staining of Rubisco (lower). YL, young leaves; NS, fully expanded no senescence mature leaves; ES, early senescence leaves, with < 25% leaf yellowing; LS, lately senescence leaves, with > 50% leaf yellowing (please also see Figure 5A). Asterisks indicate a significant difference (P ≤ 0.01).

Arabidopsis MKK4 and MKK5 function redundantly upstream of MPK3/MPK6 in several developmental processes (Xu and Zhang, 2015). In addition, expression of gain-of-function constitutively active DD form of these MAPKKs is sufficient to induce senescence. To gain loss-of-function evidence, we compared the leaf senescence in wild type, *mkk4* single, *mkk5* single, and *mkk4 mkk5* double mutants in detached leaves under continuous light. Slight delay in leaf senescence was observed in *mkk4* or *mkk5* single mutant compared to wild type (Supplementary Figure 2A). In contrast, leaf senescence was significantly delayed in the *mkk4 mkk5* double mutant (Figure 3A). Associated with the delayed phenotype, no induction of *SAG12* gene expression was detected in *mkk4 mkk5* double mutants 6 days after detachment. In wild type, the level of *SAG12* transcript was detected in 4 days after detachment and increased greatly afterward (Figure 3B). Measurement of chlorophyll content and F_*v*_/F_m_ showed that greater losses in chlorophyll content and F_*v*_/F_m_ value in the wild type plants than those in the *mkk4 mkk5* double mutant plants (Figures 3C,D). To further confirm whether MKK4 and MKK5 acted upstream of MPK3 and MPK6 during senescence, we evaluated the phosphorylation activation of MPK3 and MPK6 during different leaf senescence stage. As shown in Figure 3E, age-dependent leaf senescence-induced MPK3/MPK6 activation was impaired in the *mkk4 mkk5* double mutant. These data suggested that MKK4 and MKK5 are involved in plant leaf senescence and play redundant function in activating MPK3/MPK6 during leaf senescence.

In addition to MKK4 and MKK5, MKK7 and MKK9 were also reported to be upstream of MPK3/MPK6 (Xu et al., 2008; Zhou et al., 2009; Jia et al., 2016). As a result, we also examined the leaf senescence in *mkk7* single, *mkk9* single, and *mkk7 mkk9* double mutants under the same experimental condition. As shown in Supplementary Figure 2A, we did not see an obvious difference in leaf senescence in comparison to wild type. As a result, we conclude that MPK4/MPK5, but not MKK7/MKK9, are upstream of the MPK3/MPK6 in plant leaf senescence.

### Expression of Matrix Metalloproteinases Genes Are Highly Induced After Gain-of-Function Activation of MPK3/MPK6 and During Senescence

**FIGURE 4:**
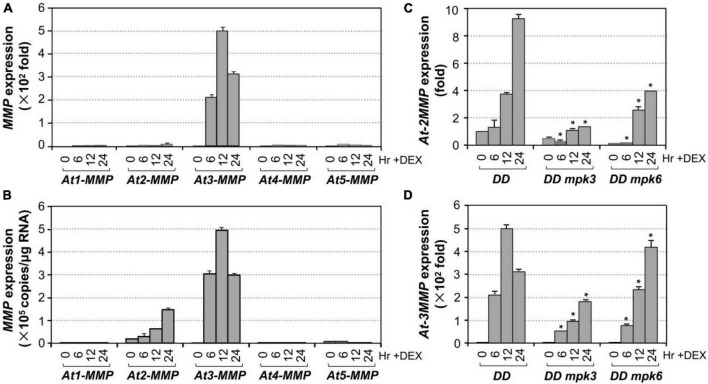
Induction of Arabidopsis *MMP* genes expression after MPK3/MPK6 activation in the gain-of-function *DD* seedlings. **(A)** The induction of *MMP* gene expression was quantified by RT-qPCR. *MMP* induction (fold of induction relative to the level before induction) was calculated using the double ΔCt method. The expression of *EF1*α was used to normalize the samples. **(B)** Absolute expression levels of *MMP* induction was calculated as copies numbers per μg of total RNA by the calibration curve established for each *MMP* gene, which allows comparison to expression levels between different *MMP* genes. Values were means ± SD, *n* = 3. **(C,D)** Activation of *At2-MMP* **(C)** and *At3-MMP* **(D)** expression in the *DD* seedlings were compromised in either *mpk3* or *mpk6* mutant background. Gene expression was quantified by RT-qPCR and calculated as in **(A)**. Asterisks indicate a significant difference (*P* ≤ 0.001). Values were means ± SD, *n* = 3.

To identify unknown components downstream of MPK3/MPK6 cascade in leaf senescence, we mined the expression profiling data in *DD* transgenic plants after DEX treatment in our previous study (Su et al., 2018). *At3-MMP* is one of the highest induced genes. As a result, we quantified the expression of all five *At-MMP* genes in *DD* plants after DEX treatment and in leaves during senescence using RT-qPCR. As shown in Figure 4A, the expression of *At2-MMP* and *At3-MMP* was induced approximately 10 and 500-folds over its basal level, respectively. In contrast, *At1-MMP*, *At4-MMP*, and *At5-MMP* transcripts could be reliably detected, but were not induced after MPK3/MPK6 activation. To eliminate the influence of amplification efficiency during qPCR and better compare the expression levels of different *MMP* genes, we also generated the standard curves and calculated the absolute expression levels of each *MMP* after activation of *DD*. As shown in Figure 4B, we found that the expression levels of *At2-MMP* and *At3-MMP* were among the highest, while *At1-MMP*, *At4-MMP*, and *At5-MMP* were expressed at low levels. To confirm that MPK3 and MPK6 were responsible for the induction of *At2-MMP* and *At3-MMP* expression level in the *DD* plants, we also examined the expression of *At2-MMP* and *At3-MMP* in *DD*, *DD mpk3*, and *DD mpk6* plants. As shown in Figures 4C,D, while *At2-MMP* and *At3-MMP* expression was highly induced in *DD* plants after DEX treatment, their expression in *DD mpk3* and *DD mpk6* plants was partially compromised, correlating with the delayed leaf senescence (Figure 2A). This finding demonstrated that the induction of *At2-MMP* and *At3-MMP* in *DD* seedlings after DEX treatment is a result of MPK3/MPK6 activation.

**FIGURE 5:**
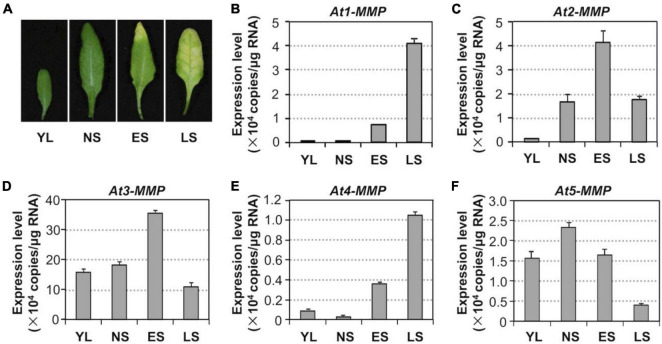
Activation of *MMP* expression during leaf senescence in *Arabidopsis.* **(A)** Leaf senescence in wild type plants at four different developmental stages. YL, young leaves; NS, fully expanded no senescence mature leaves; ES, early senescence leaves, with < 25% leaf yellowing; LS, lately senescence leaves, with > 50% leaf yellowing. **(B–F)** Transcript levels of *MMPs* increase in an age-dependent manner. Samples were collected from leaves shown in **(A)** at the indicated developmental stages. Gene expression was quantified by RT-qPCR and calculated as copy numbers per μg of total RNA. Values are means ± SD, *n* = 3.

To assess whether *MMP* genes are involved in regulating leaf senescence, we first investigated their expression patterns during the senescence process (Figure 5A). RT-qPCR analysis revealed that the levels of all *MMPs* except *At5-MMP* transcripts increased during the progression of leaf development and senescence (Figures 5B–F). *At3-MMP* had the highest copy numbers, approximately 10 times more than the other four homologs during senescence (Figure 5D). Based on these results, we conclude that Arabidopsis *MMPs* are senescence-associated genes (*SAGs*) and might be involved in regulating leaf senescence downstream of MPK3/MPK6 cascade.

### Both Constitutive and Inducible Overexpression of *At-3MMP* Accelerates Leaf Senescence

**FIGURE 6:**
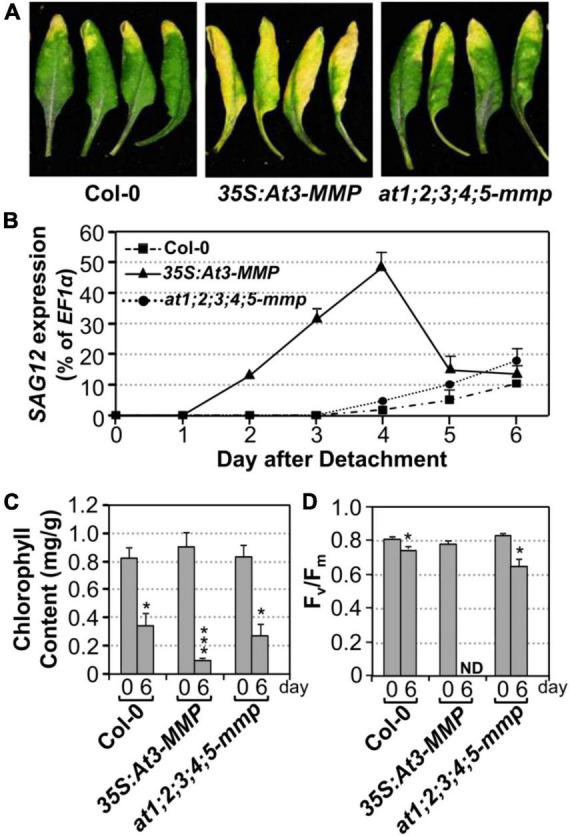
Constitutive overexpression of *At3-MMP* leads to leaf senescence under continuous light. **(A)** Fully expanded leaves from 4-week-old soil-grown plants were detached and their petioles were inserted into 0.6% agar medium. Photos were taken after detachment 6 days. **(B)** RT-qPCR analysis of the transcript levels of *SAG12* in the leaves of plants shown in **(A)** at indicated times. The expression of *EF1*α was used as an internal reference. Values are means ± SD, *n* = 3. **(C,D)** Chlorophyll content **(C)** and F_*v*_/F_m_ **(D)** in leaves shown in **(A)**. Values are means ± SD, *n* = 10. ND, not detectable. Asterisks indicate a significant difference (* P < 0.05, *** P ≤ 0.001).

Next, we tested whether an elevated expression of *At3-MMP* is sufficient to accelerate leaf senescence. Transgenic plants overexpressing a full-length *At3-MMP* gene with a C-terminal double HA tag driven by the constitutive 35S promoter (*35S:At3-MMP-dHA*) were generated. Two independent transgenic lines (#15 and #131) with different expression levels based on immunoblot analysis were selected for further analyses. We found that *35S:At3-MMP-dHA* plants exhibited an early senescence phenotype compared with Col-0 (Supplementary Figure 3A). Next, we characterized the senescence of single leaves at different ages. As shown in Supplementary Figure 3B, the oldest seven leaves (numbered from the bottom; the first leaf is the oldest and the 12th leaf is the youngest) of the *35S:At3-MMP-dHA* plants showed senescence, while only three leaves of the wild-type plants senesced. In addition, the cauline leaves of *35S:At3-MMP-dHA* plants turned yellow earlier than the wild-type counterparts (Supplementary Figure 3C). Furthermore, there was a good correlation between the expression levels of At-3MMP protein and the severity of leaf senescence in different transgene lines (Supplementary Figures 4A,B). Clear acceleration of leaf senescence was also observed when the fully expanded leaves were excised from *35S:At3-MMP-dHA* plants (Figure 6A). We also monitored the expression of *SAG12*, chlorophyll content, and F_*v*_/F_m_. As shown in Figure 6B, quicker/earlier *SAG12* induction was associated with the early senescence symptoms in *35S:At3-MMP-dHA* plants. The *SAG12* transcript levels were induced significantly from 2 days in the *35S:At3-MMP-dHA* plants. In contrast, the induction of *SAG12* expression was undetectable in the first 4 days in wild type and only increased slightly afterward. Similarly, more severe reduction in chlorophyll content and F_*v*_/F_m_ value happened in *35S:At3-MMP-dHA* plants (Figures 6C,D). Taken together, these results suggested that constitutive overexpression of *At-3MMP* is sufficient to lead to early leaf senescence.

**FIGURE 7:**
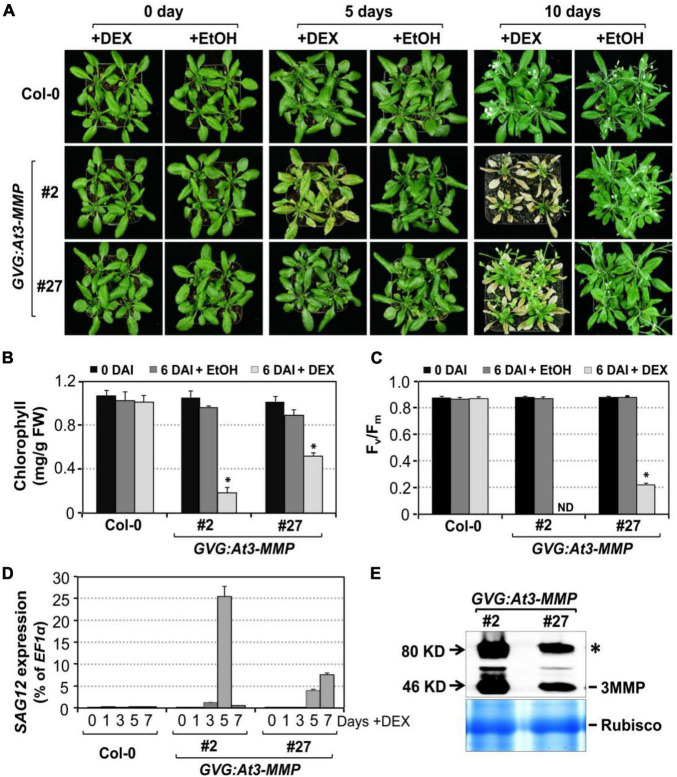
Conditional expression of *At-3MMP* is sufficient to induce leaf senescence. **(A)** The senescence phenotype of inducible *At3-MMP* transgenic lines (#2 and #27) and wild type plants after treatment with 30 μM DEX or EtOH (solvent control) for 0, 5, and 10 days. 4-week-old soil-grown plants were sprayed with DEX or EtOH. Photos were taken at the indicated time points. **(B,C)** Chlorophyll content **(B)** and F_*v*_/F_m_ **(C)** of fifth to seventh leaves in wild-type and transgenic plants before and after DEX or EtOH treatment. Values are means ± SD, *n* = 10. ND: no detected. **(D)** RT-qPCR analysis of *SAG12* transcript levels at indicated times after DEX treatment. Values are means ± SD, *n* = 3. **(E)** Induction of At3-MMP protein at 24 h after DEX (5 μM) treatment. HA-tagged At3-MMP protein was detected by immunoblot analysis using anti-HA antibody (upper). Coomassie brilliant blue staining of a duplicated gel was used to confirm equal loading (bottom). Asterisks indicate a significant difference (P ≤ 0.001).

To further confirm the role of Arabidopsis *MMP*s in leaf senescence and avoid possible secondary complications associated with constitutive overexpression, we also performed a gain-of-function study using the glucocorticoid-inducible system to direct the expression of *3MMP-dHA* transgene (*GVG:At3-MMP-dHA*). Two independent transgenic lines (#2 and #27) that accumulated At-3MMP protein at different levels were identified by immunoblot analysis and the T3 homozygous plants were selected for further experiments. We induced *At-3MMP* expression by treating 4-week-old plants with 30 μM DEX. As shown in Figure 7A, obvious leaf yellowing and leaf death were observed in the *GVG: At3-MMP-dHA* transgenic line #2 and #27 at 5 and 10 days, respectively. In contrast, no leaf senescence was observed in the wild type plants. No leaf senescence was detected in EtOH-treated control plants either. Associated with the leaf yellowing, chlorophyll and F_*v*_/F_m_ loss and *SAG12* gene expression were readily detectable in DEX-treated *GVG: At3-MMP-dHA* plants, but not solvent control-treated plants or wild type plants (Figures 7B–D). Immunoblot analysis showed that At3-MMP protein level was higher in line #2 compared to line #27 (Figure 7E). Correlating with the induction level, line #2 leaves became fully senescent or died, while line #27 leaves were still partially green (Figure 7A). Taken together, overexpression of *At-3MMP*, either constitutively under the control of 35S promoter or conditionally under the control of a steroid-inducible promoter, is sufficient to promote leaf senescence.

### High-Order *mmp* Mutant Fails to Show Delayed Leaf Senescence

To provide loss-of-functional evidence to support the role of *At-MMPs* in leaf senescence, we obtained loss-of-function T-DNA insertion mutants of all five Arabidopsis *MMP* genes from ABRC. Semi-quantitative RT-PCR analyses showed that *At-MMPs* transcripts were not detectable in the senescing leaves of the mutants, suggesting that these lines were all null knockout mutants (Supplementary Figure 5). No obvious growth/developmental or senescence phenotype was observed in *at1-mmp* to *at5-mmp* single mutant, *mmp2 mmp3*, *mmp3 mmp5* double mutants, and *mmp2 mmp3 mmp5* triple mutants (Supplementary Figure 6). Phylogenetic analysis revealed that all five Arabidopsis *MMP* genes shared high homology (Supplementary Figure 7). As a result, we generated pentuple *at1;2;3;4;5-mmp* mutant. Under our growth conditions, the *at1;2;3;4;5-mmp* high-order mutant was still wild-type like and no delayed senescence was observed (Supplementary Figure 6). We also examined the senescence of detached leaves of wild type and *at1;2;3;4;5-mmp* higher-order mutant under continuous light and found that there was no obvious difference between them in leaf yellowing, *SAG12* gene activation, chlorophyll content, and F_*v*_/F_m_ (Figure 6). Taken together, we conclude that although expression of *At3-MMP* is sufficient to induce leaf senescence, loss of *MMP*s is not sufficient to block leaf senescence, suggesting the presence of other redundant pathway(s) that are sufficient to signal/execute leaf senescence in the absence of *MMPs*.

## Discussion

In this study, we demonstrate that MPK3/MPK6, and their upstream MAPKKs, MKK4, and MKK5, are involved in regulating leaf senescence in *Arabidopsis* (Figures 1–3). Four of the five MMP family members are highly induced during leaf senescence (Figure 5), qualifying them as senescence-associated genes (*SAGs*). Among them, *At3-MMP* is induced to the highest level. Interestingly, *At3-MMP* is one of the top differentially expressed genes in *DD* plants (Su et al., 2018). More detailed qPCR analyses demonstrated that the expression of *At-3MMP* and, to a lesser extent, *At-2MMP* is rapidly induced by MPK3/MPK6 activation (Figure 4). Either constitutive or inducible overexpression of *At3-MMP* is sufficient to trigger leaf senescence (Figures 6, 7 and Supplementary Figure 3), suggesting a linear pathway from MPK3/MPK6 MAPK cascade to *MMP* target genes in leaf senescence.

Arabidopsis MPK6, along with MKK9, a MAPKK, has been implicated in leaf senescence in Arabidopsis (Zhou et al., 2009). It was reported that senescence is delayed in detached leaves of both *mpk6* and *mkk9* single mutant plants, while overexpression of *MKK9* accelerates premature senescence in leaves. In this study, we tested both *mpk3* and *mpk6* single mutants. As shown in Supplementary Figure 2A, neither showed a clear senescence phenotype. We failed to observe any senescence phenotype in the chemical genetically rescued *MPK3SR* and *MPK6SR* double mutant either (Supplementary Figure 2B). The genotypes of *MPK3SR* and *MPK6SR* double mutant plants are *mpk3 mpk6 proMPK3:MPK3*^TG^** and *mpk3 mpk6 proMPK6:MPK6*^YG^**, respectively. In the presence of 4-amino-1-tert-butyl-3-(1′-naphthyl) pyrazolo [3,4-d] pyrimidine (NA-PP1), the activity of chemical-sensitized MPK3*^TG^* or MPK6*^YG^* is inhibited, making the *MPK3SR* and *MPK6SR* activity null *mpk3 mpk6* double mutants (Xu et al., 2014; Su et al., 2017). The failure in observation of any senescence phenotype of *MPK3SR* and *MPK6SR* double mutant plants is likely because NA-PP1 can be metabolized in plant cells rather quickly, which makes it difficult to maintain a MPK3/MPK6 activity null condition for an extended period of time (over several days) to inhibit senescence. In contrast, we consistently observed a delayed senescence in *mkk4 mkk5* double mutant leaves (Figure 3). In this double mutant, the *mkk4* tilling allele has a substitution of the conserved Pro residue at the 240 position of the catalytic domain by Ser residue (CCT to TCT) and the mutated kinase carries ∼10% of the residual kinase activity. The *mkk5* tilling allele is a null mutant with a premature stop codon (Arg72 to opal; CGA to TGA). As a result, the *mkk4 mkk5* double mutant has about 5% of the residual activity if MKK4 and MKK5 contribute equally to activate downstream MPK3/MPK6 (Su et al., 2017). In addition to Arabidopsis MKK4 and MKK5 (two plant Group C MAPKKs), Arabidopsis MKK7 and MKK9, two members sharing closest homolog in the Group D MAPKKs (Ichimura et al., 2002), were also reported to be upstream of MPK3 and/or MPK6 (Xu et al., 2008; Jia et al., 2016). However, we did not observe any obvious senescence phenotype in *mkk7* single, *mkk9* single, and *mkk7 mkk9* double mutants (Supplementary Figure 2A). As a result, we conclude that MKK4/MKK5, but not MKK7/MKK9, are upstream of the MPK3/MPK6 in plant leaf senescence.

In the gain-of-function *DD* transgenic system, our previous study showed that *DD* transgene expression is detectable within 2 h and HR-like cell death appears ∼24 h after the application of DEX. The dead leaves stay green and became brittle, similar to HR cell death triggered by pathogens (Ren et al., 2002). In this study, we confirm that strong and prolonged MPK3/MPK6 activation causes HR-like cell death in *DD* leaves after treatment with higher concentrations of DEX (>100 nM). In contrast, when *DD* leaves are treated with lower concentrations of DEX (<100 nM), leaf senescence is induced, which is associated with lower levels of MPK3/MPK6 phosphorylation and activation (Figure 1 and Supplementary Figure 1). These results provide another piece of evidence to support the hypothesis that MAPK signaling strength/duration could be important to the biological outcomes. Based on genetic evidence, we concluded that different developmental processes may have differential signal thresholds (strength and/or duration), which could specify the MAPK function in a quantitative way (Wang et al., 2007, 2008; Xu and Zhang, 2015).

Unlike mammalian *MMP* genes, which have been studied in great detail, the functions of *MMPs* in plants are mostly unknown. Based on the gene expression pattern, *SMEP1*, a plant matrix metalloproteinase gene isolated from soybean, was found to be expressed in mature leaves but not in young leaves and tissues (Pak et al., 1997). In cucumber, the *Cs1-MMP* was expressed in leaves during senescence and may be involved in programmed cell death (Delorme et al., 2000). In this study, we demonstrate that *At-MMPs* are *SAGs*, and play an important role in leaf senescence. Four of the five *MMP* transcripts are induced during the progression of leaf senescence, especially *At3-MMP*, which shows the greatest induction (Figure 5). Furthermore, constitutive or inducible overexpression of *At3-MMP* is sufficient to induce leaf senescence, as indicated by a loss/reduction in chlorophyll and photochemical efficiency, and the induction of *SAG12* gene expression (Figures 6, 7 and Supplementary Figure 3). Collectively, these data suggest that plant *MMPs* are involved in regulating leaf senescence.

We obtained T-DNA insertion mutants of all five *MMP* genes from ARBC. Semi-quantitative PCR analysis confirms that all of them are complete knock-out mutants (Supplementary Figure 5). However, we failed to observe a difference in leaf senescence between wild type and the single, double, triple, and pentuple *mmp* mutants (Figure 6 and Supplementary Figure 6), suggesting parallel pathway(s) that is sufficient to execute leaf senescence. Transcriptome analyses of leaf senescence identified thousands of *SAGs*. However, most *SAG* mutants do not have altered leaf senescence, probably due to functional redundancy or the lack of a pronounced effect on leaf senescence (He et al., 2001). A large number of plant signaling molecules including plant hormones, kinases including receptor-like protein kinases, and transcription factors contribute to the regulation and execution of senescence at the cellular and subcellular levels, suggesting functionally redundant pathways (He et al., 2001; Guo et al., 2004; Lim et al., 2007; Schippers et al., 2007; Kim H. J. et al., 2016; Kim J. et al., 2016). Recently, MAPKKK18 was identified as a positive regulator of leaf senescence. It controls the timing of leaf senescence via its kinase activity in an ABA-dependent manner (Matsuoka et al., 2015). Phylogenetic analysis revealed that MAPKKK18 belongs to a different subgroup of MAPKKKs as YDA, MAPKKK3, and MAPKKK5, three MAPKKKs that have been functionally placed upstream of the MKK4/MKK5-MPK3/MPK6 module in various biological processes (Bergmann et al., 2004; Wang et al., 2007; Meng et al., 2012; Bi et al., 2018; Sun et al., 2018; Supplementary Figure 8). MEKK1, a MAPKKK that belongs to yet another subgroup of MAPKKKs, was reported to regulate leaf senescence by directly phosphorylating the WRKY53, a senescence-promoting transcription factor (Miao et al., 2007). At this stage, whether MAPKKK18 and/or MEKK1 are in the same MAPK cascade as MKK4/MKK5 and MPK3/MPK6 in leaf senescence is unknown.

In *35S:At3-MMP-HA* and *GVG:At3-MMP-HA* transgenic plants, we detected two HA-tagged protein bands with molecular masses of 46 and 80 kDa by immunoblot analysis (Figure 7 and Supplementary Figure 4). The theoretical mass of At3-MMP based on its amino acid sequence is ∼43 kDa. A similar finding was reported in a previous study, in which the larger protein band was speculated to be glycosylated or dimer form of MMP protein (Schiermeyer et al., 2009). Substrate identification will provide further insights into how MMPs carry out their function(s) in plant leaf senescence, an important part of plant growth/development that is influenced by a wide variety of internal and environmental factors. Leaf senescence normally occurs at the final stage of the plant growth season or life cycle, and is essential for nutrient recycling and crop yield (Gan, 2003; Lim et al., 2007). In contrast, premature leaf senescence, as an exit strategy when plants are confronted with biotic/abiotic stresses, may reduce yield in crop by limiting the growth stage and causing post-harvest spoilage. As a result, understanding the regulation of leaf senescence will not only reveal insights into this fundamental developmental process but also shed light on ways of manipulating senescence for agriculture application.

## Data Availability Statement

Publicly available datasets were analyzed in this study. This data can be found here: NCBI Sequence Read Archive (SRP111959).

## Author Contributions

HW performed most of the experiments and analyzed the data. QS, JL, and LY provided technical assistance. HW, SZ, and JX conceived the project, designed the experiments, and wrote the manuscript. JX served as the author responsible for contact and ensure communication. All authors contributed to the article and approved the submitted version.

## Conflict of Interest

The authors declare that the research was conducted in the absence of any commercial or financial relationships that could be construed as a potential conflict of interest.

## Publisher’s Note

All claims expressed in this article are solely those of the authors and do not necessarily represent those of their affiliated organizations, or those of the publisher, the editors and the reviewers. Any product that may be evaluated in this article, or claim that may be made by its manufacturer, is not guaranteed or endorsed by the publisher.
