# Supplementary material for: Regulation of Arabidopsis Matrix Metalloproteinases by Mitogen-Activated Protein Kinases and Their Function in Leaf Senescence
Source: Front Plant Sci. 2022 Apr 8;13:864986. doi: 10.3389/fpls.2022.864986 (PMC9024413; doi:10.3389/fpls.2022.864986)
Supplement: Supplementary file 1 [file Table_1.pdf]

Supplemental Table 1. Primers used in this study

**T-DNA insertion mutant identification**		
at1-mmp (SALK_205145C)	LP	TTCACCCGACGAACCAACTT
	RP	TGCCACGTCATCAACCGTAA
at2-mmp (SM_3_5305)	LP	TGAACGTGACTAAGACCAATAGATG
	RP	TCAAGGGTTCACACACAAGAAC
at3-mmp (SM_3_28404)	LP	AAATGACCTCTGCTTTCTCCAA
	RP	AGGAATGGCCTAACCCTAGAAG
at4-mmp (GABI_075C07)	LP	GAAGCTCGAAATCCATCTCAAT
	RP	TCGGCTAGATTTGTCGAAGTTT
at5-mmp (SAIL_390_c06)	LP	GCTTCATAAAACAAACACACCGAC
	RP	GACCATGACCCCAAATCGTTAC
LBb1.3 (SALK line)	ATTTTGCCGATTTCGGAAC	
JL202 (GABI line)	CATTTTATAATAACGCTGCGGACAT	
Spm32 (SM line)	TACGAATAAGAGCGTCCATTTTAGAGTGA	
LB3 (SAIL line)	TTCATAACCAATCTCGATACAC	
**3MMP construct**		
LP1	TCTCTTTTGGATGGTGAGGATT	
RP1	GCATGAATGAAGAACCAATCTAGG	
LP2	ATGGTGAGGATTTGTGTTTTCATC	
RP2	CACTAAATACAAAAATAATCCAAATATAATCC	
**Gene expression assay by qPCR assay**		
At1-MMP	LP	AGATCGGTCACTTGTTGGGA
	RP	TGCGTTAGTGAATCCAACCG
At2-MMP	LP	GACAGGGAAACGTAAGGTTGAC
	RP	AGTGTCTCGCTGGTGTTTAGTG
At3-MMP	LP	TCATGTATCCAACCATCAGGAC
	RP	CTTCTACCAGGAGCACCAGAAT
At4-MMP	LP	CTAGTGTGGCCGTCGATTTG

	RP	AGACTGTACTCCAACGACGT
At5-MMP	LP	CTAGGTCATTCGTCGGTGGA
	RP	TGACTCTCACGGCTAGGTTT
EF1α	LP	GATTGCCACACCTCTCACATT
	RP	CATACCAGCGTCACCATTCTT
SAG12	LP	GGATGTCCCGGTTAATGATG
	RP	TCCACTTTCTCCCCATTTTG
