# Supplementary material for: Regulation of Arabidopsis Matrix Metalloproteinases by Mitogen-Activated Protein Kinases and Their Function in Leaf Senescence
Source: Front Plant Sci. 2022 Apr 8;13:864986. doi: 10.3389/fpls.2022.864986 (PMC9024413; doi:10.3389/fpls.2022.864986)
Supplement: Supplementary file 2 [file Image_1.pdf]

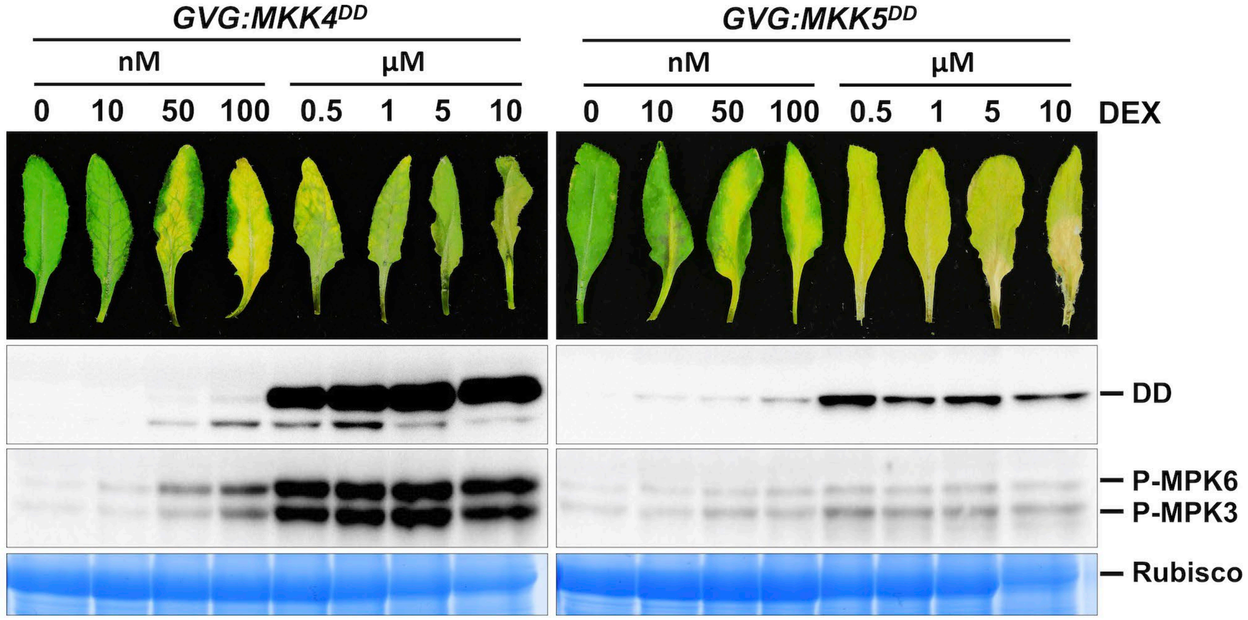

Supplemental Figure 1. Weak induction of constitutively active MAPKK promotes leaf senescence.

Treatment of *AtMKK4*^{DD} and *AtMKK5*^{DD} transgenic plants with different concentrations of DEX can control the amplitude of MPK3/MPK6 activation. Fully expanded leaves from 4-week-old soil-grown plants were detached and their petioles were inserted into 0.6% agar medium with different concentrations of DEX. Photos were taken at 3 days after DEX treatment (first panel). Note: leaves treated with DEX at 0.5 µM or above showed rapid cell death and stayed green. Leaf samples were collected at 1 day after DEX treatment. DD protein induction was detected by immunoblot assay using an anti-flag antibody (second panel). MAPK activation was detected by immunoblot assay using an anti-pTEpY antibody (third panel). Coomassie brilliant blue (CBB) staining of duplicate gels was used to show equal loading (fourth panel)
