# Supplementary material for: Regulation of Arabidopsis Matrix Metalloproteinases by Mitogen-Activated Protein Kinases and Their Function in Leaf Senescence
Source: Front Plant Sci. 2022 Apr 8;13:864986. doi: 10.3389/fpls.2022.864986 (PMC9024413; doi:10.3389/fpls.2022.864986)
Supplement: Supplementary file 3 [file Image_2.pdf]

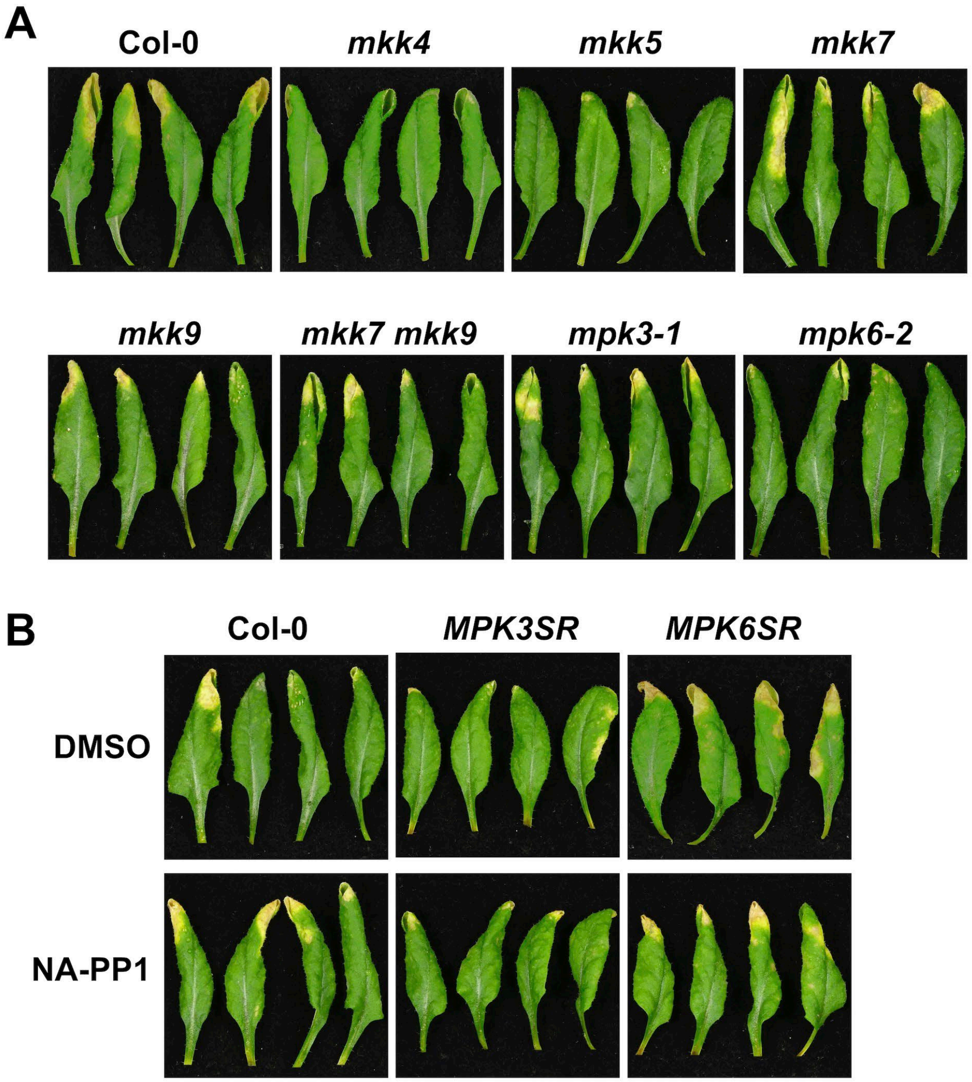

Supplemental Figure 2. Leaf senescence in Col-0, *mpk3*, *mpk6*, *mkk4*, *mkk5*, *mkk7*, *mkk9*, *mkk7 mkk9*, *MPK3SR*, and *MPK6SR* plants.

Fully expanded leaves from 4-week-old soil-grown plants were detached and their petioles were inserted into 0.6% agar medium under continuous light. *MPK3SR* and *MPK6SR* were pretreated with DMSO (solvent) or NA-PP1 (10 μM) for 3 h and their petioles were inserted into 0.6% agar medium with DMSO or 10 μM NA-PP1. Photos were taken 6 days after detachment.
