# Supplementary material for: Regulation of Arabidopsis Matrix Metalloproteinases by Mitogen-Activated Protein Kinases and Their Function in Leaf Senescence
Source: Front Plant Sci. 2022 Apr 8;13:864986. doi: 10.3389/fpls.2022.864986 (PMC9024413; doi:10.3389/fpls.2022.864986)
Supplement: Supplementary file 4 [file Image_3.pdf]

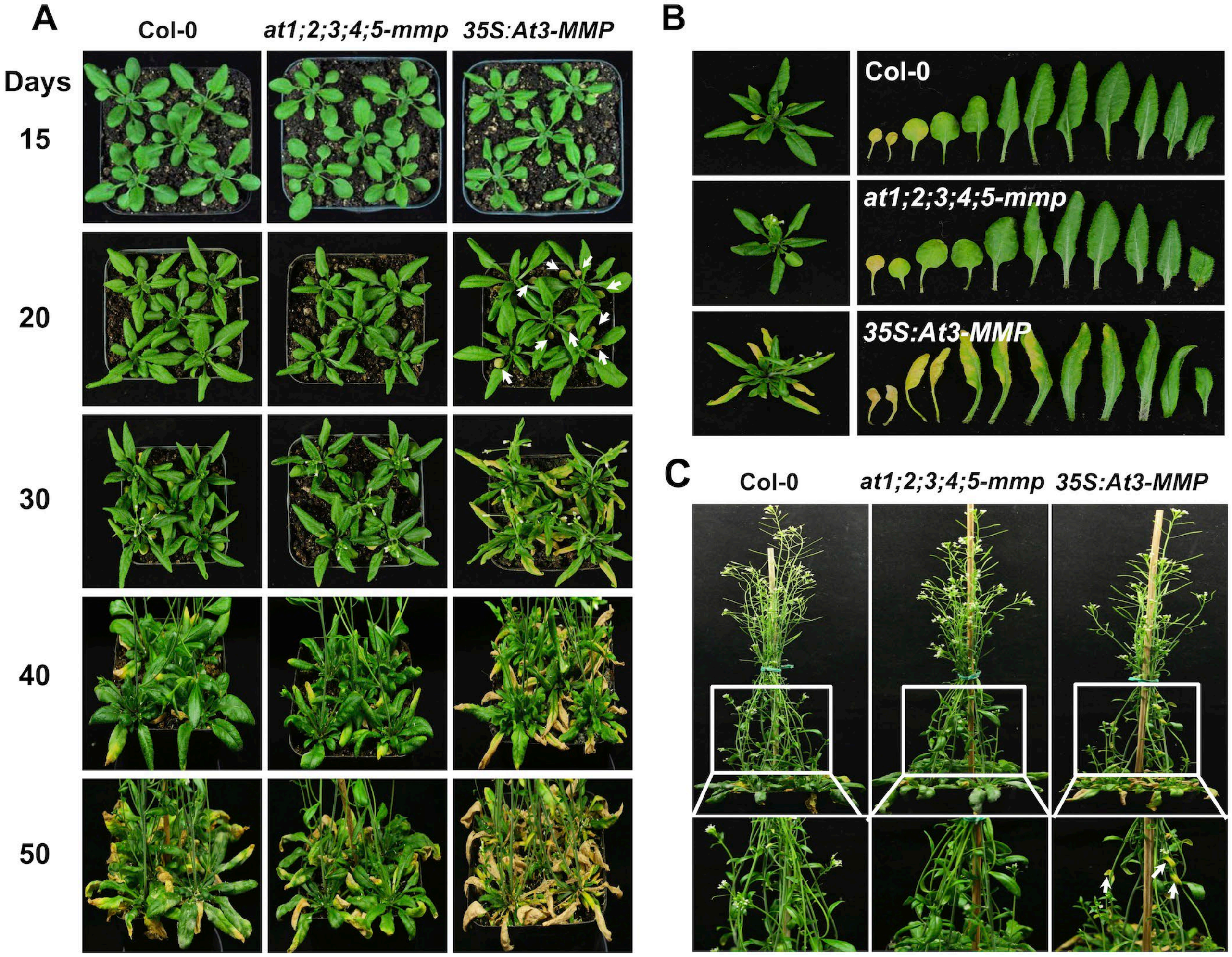

Supplemental Figure 3. Constitutive overexpression of *At3-MMP* triggers leaf senescence. (**A**) The age-dependent senescence phenotype of wild type, *at1;2;3;4;5-mmp* high-order mutant, and *35S:At3-MMP* transgenic plants. The plants were grown in soil under long-day conditions side by side. Photos were taken from 15 to 50 days after seed germination. White arrows indicate yellowing leaves. (**B**) Senescence phenotype of 4-week-old Col-0, *35S:At3-MMP*, and *at1;2;3;4;5-mmp* high-order mutant plants. The leaves were detached and arranged according to their age when the images were taken. (**C**) The levels of cauline leaf senescence in wild type, *mmp1;2;3;4;5* high-order mutant, and *35S:At3-MMP* plants. Photos were taken 40 days after germination. White arrows indicate yellowing leaves.
