# Supplementary material for: Regulation of Arabidopsis Matrix Metalloproteinases by Mitogen-Activated Protein Kinases and Their Function in Leaf Senescence
Source: Front Plant Sci. 2022 Apr 8;13:864986. doi: 10.3389/fpls.2022.864986 (PMC9024413; doi:10.3389/fpls.2022.864986)
Supplement: Supplementary file 5 [file Image_4.pdf]

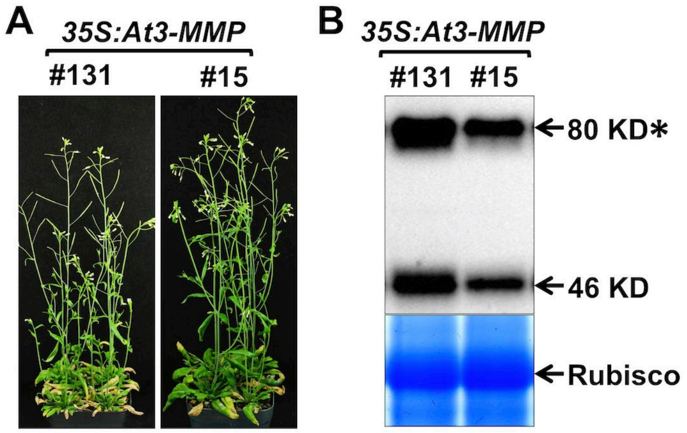

Supplemental Figure 4. Transgenic line with higher At3-MMP protein expression shows more severe leaf senescence.

(**A**) Transgenic lines with different levels of At3-MMP protein show different severity of leaf senescence. (**B**) Expression levels of *At3-MMP* transgene were determined by Immunoblot assay. The anti-HA antibody was used for detecting the levels of At-3MMP protein (upper panel). Coomassie brilliant blue staining of a duplicated gel was shown to confirm equal loading (lower panel).
