# Supplementary material for: Regulation of Arabidopsis Matrix Metalloproteinases by Mitogen-Activated Protein Kinases and Their Function in Leaf Senescence
Source: Front Plant Sci. 2022 Apr 8;13:864986. doi: 10.3389/fpls.2022.864986 (PMC9024413; doi:10.3389/fpls.2022.864986)
Supplement: Supplementary file 6 [file Image_5.pdf]

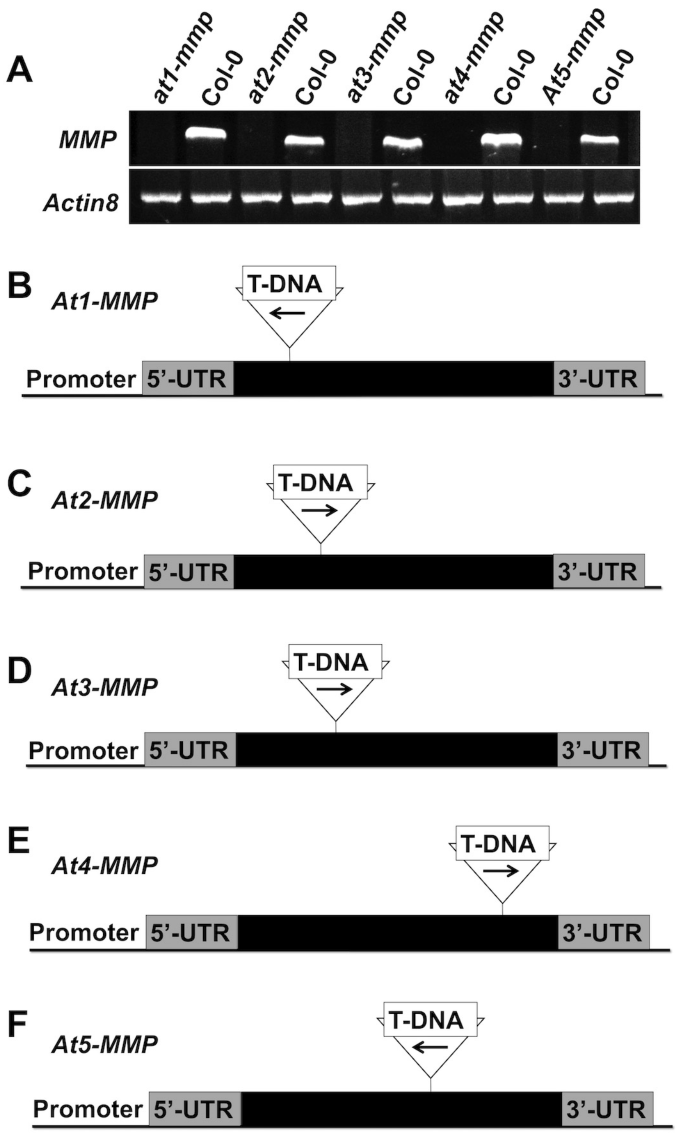


**Supplemental Figure 5. The T-DNA insertion alleles of *mmp* mutants**
(**A**) RT-qPCR analysis of *MMP* transcript levels in Col-0 and the *mmp* mutants. RNA was isolated from the senescing leaves. After reverse transcription, full length cDNA regions were detected by semi-quantitative PCR. (**B**) to (**F**) Diagrams showing the positions of T-DNA insertion in the *mmp* mutant alleles.
