# Supplementary material for: Regulation of Arabidopsis Matrix Metalloproteinases by Mitogen-Activated Protein Kinases and Their Function in Leaf Senescence
Source: Front Plant Sci. 2022 Apr 8;13:864986. doi: 10.3389/fpls.2022.864986 (PMC9024413; doi:10.3389/fpls.2022.864986)
Supplement: Supplementary file 7 [file Image_6.pdf]

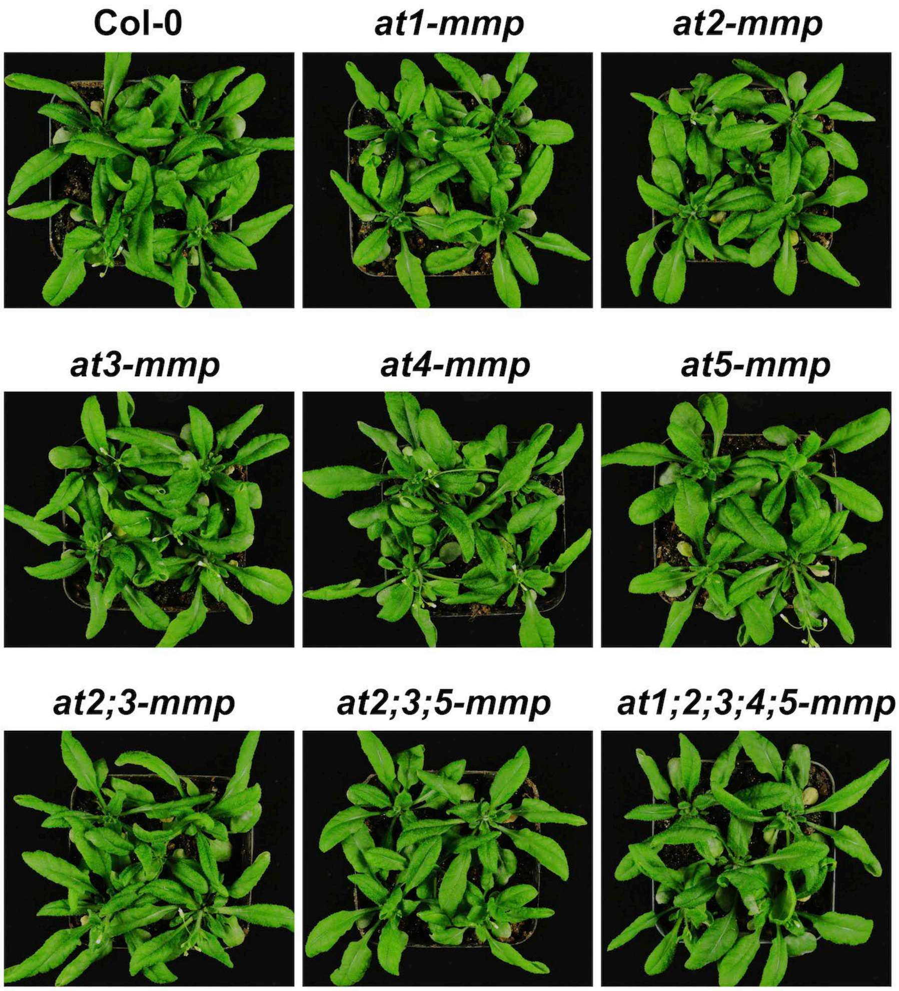

Supplemental Figure 6. The single, double, triple, and quintuple *mmp* mutants show a normal growth rate as the wild type.

The plants were grown in soil under long-day conditions side by side. Photos were taken three weeks after germination.
