# Supplementary material for: Regulation of Arabidopsis Matrix Metalloproteinases by Mitogen-Activated Protein Kinases and Their Function in Leaf Senescence
Source: Front Plant Sci. 2022 Apr 8;13:864986. doi: 10.3389/fpls.2022.864986 (PMC9024413; doi:10.3389/fpls.2022.864986)
Supplement: Supplementary file 8 [file Image_7.pdf]

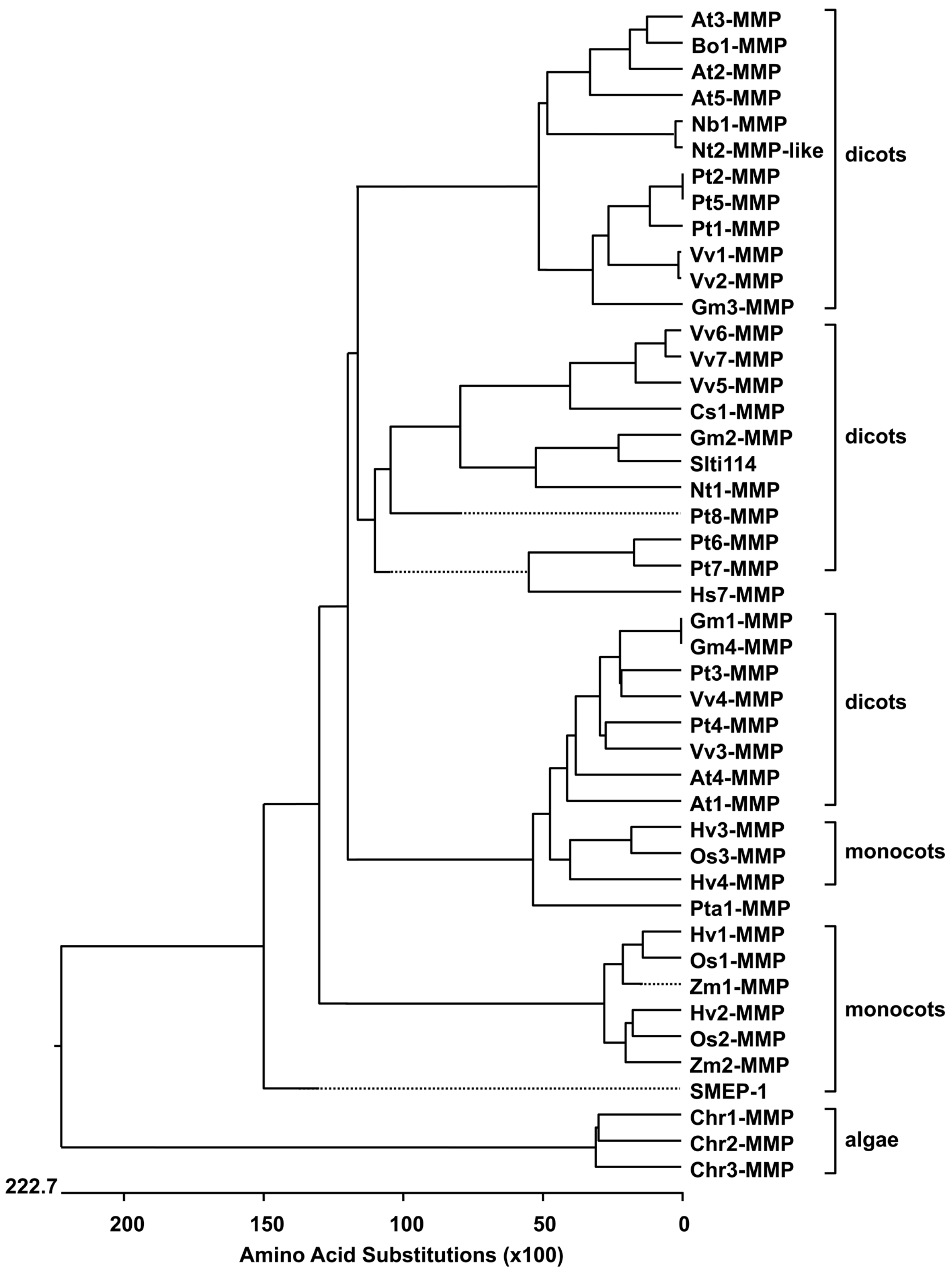

Supplemental Figure 7. Phylogenetic analysis of plant MMPs.

The full-length amino acid sequences were aligned using the Clustal W method, and the phylogenetic tree was generated using DNAstar.
